# Cathepsin S provokes interleukin-6 (IL-6) trans-signaling through cleavage of the IL-6 receptor in vitro

**DOI:** 10.1038/s41598-020-77884-4

**Published:** 2020-12-10

**Authors:** Charlotte M. Flynn, Yvonne Garbers, Stefan Düsterhöft, Rielana Wichert, Juliane Lokau, Christian H. K. Lehmann, Diana Dudziak, Bernd Schröder, Christoph Becker-Pauly, Stefan Rose-John, Samadhi Aparicio-Siegmund, Christoph Garbers

**Affiliations:** 1 Institute of Biochemistry, Kiel University, Kiel, Germany; 2 Institute of Psychology, Kiel University, Kiel, Germany; 3 Institute of Molecular Pharmacology, RWTH Aachen University, Aachen, Germany; 4 Department of Pathology, Medical Faculty, Otto-Von-Guericke-University Magdeburg, Magdeburg, Germany; 5 Laboratory of Dendritic Cell Biology, Department of Dermatology, University Hospital Erlangen, Friedrich-Alexander University of Erlangen-Nürnberg, Erlangen, Germany; 6 Deutsches Zentrum Immuntherapie (DZI) and Medical Immunology Campus Erlangen, Erlangen, Germany; 7 Institute for Physiological Chemistry, Medizinisch-Theoretisches Zentrum MTZ, Technische Universität Dresden, Fiedlerstraße 42, 01307 Dresden, Germany

**Keywords:** Biochemistry, Cytokines, Proteases

## Abstract

The cytokine interleukin-6 (IL-6) fulfills its pleiotropic functions via different modes of signaling. Regenerative and anti-inflammatory activities are mediated via classic signaling, in which IL-6 binds to the membrane-bound IL-6 receptor (IL-6R). For IL-6 trans-signaling, which accounts for the pro-inflammatory properties of the cytokine, IL-6 activates its target cells via soluble forms of the IL-6R (sIL-6R). We have previously shown that the majority of sIL-6R in human serum originates from proteolytic cleavage and mapped the cleavage site of the IL-6R. The cleavage occurs between Pro-355 and Val-356, which is the same cleavage site that the metalloprotease ADAM17 uses in vitro. However, sIL-6R serum levels are unchanged in hypomorphic ADAM17^ex/ex^ mice, making the involvement of ADAM17 questionable. In order to identify other proteases that could be relevant for sIL-6R generation in vivo, we perform a screening approach based on the known cleavage site. We identify several candidate proteases and characterize the cysteine protease cathepsin S (CTSS) in detail. We show that CTSS is able to cleave the IL-6R in vitro and that the released sIL-6R is biologically active and can induce IL-6 trans-signaling. However, CTSS does not use the Pro-355/Val-356 cleavage site, and sIL-6R serum levels are not altered in *Ctss*^−/−^ mice. In conclusion, we identify a novel protease of the IL-6R that can induce IL-6 trans-signaling, but does not contribute to steady-state sIL-6R serum levels.

## Introduction

Interleukin-6 (IL-6) is the founding and name-giving member of the IL-6 family of cytokines^1^. In healthy humans, IL-6 serum levels are in the low pg/ml range or even undetectable. However, during inflammatory events, IL-6 levels rise dramatically and can even reach amounts of several µg/ml in patients with sepsis^2^. The involvement of IL-6 in practically all human inflammatory diseases has made the cytokine an important therapeutic target, and antibodies targeting either IL-6 or the IL-6 receptor (IL-6R) are used in the clinics, e.g. for the treatment of rheumatoid arthritis, cytokine release syndrome and Castleman disease^3,4^.

IL-6 activates its target cells by binding to the IL-6 receptor (IL-6R), which is mainly expressed on hepatocytes and different leukocyte subsets like T cells, B cells, megakaryocytes and neutrophils^5^. The formation of the IL-6/IL-6R complex induces homodimerization of the common β-receptor glycoprotein (gp)130 and subsequently the activation of several intracellular signaling cascades, e.g. the Janus kinase/Signal Transducer and Activator of Transcription (Jak/STAT), the phosphoinositide 3-kinase (PI3K) and the mitogen-activated protein kinase (MAPK) pathways^6^. This mode of signaling has been termed classic signaling and is thought to be responsible for the regenerative, anti-inflammatory properties of IL-6^3,7^.

Besides classic signaling, IL-6 can also bind to and signal via soluble variants of the IL-6R (sIL-6R). The resulting IL-6/sIL-6R complex activates cells via gp130 homodimerization in an agonistic manner and by this mechanism significantly expands the number of cell types that can be activated by IL-6, as gp130 is expressed ubiquitously and IL-6 does not require membrane-bound IL-6R expression. This so-called trans-signaling is responsible for the pro-inflammatory actions of the cytokine^8,9^. In healthy humans, sIL-6R serum levels are in the range of 20–80 ng/ml^10^. A single nucleotide polymorphism (rs2228145), which results in the exchange of an asparagine into an alanine residue at position 358 of the IL-6R protein in close proximity of the ADAM17 cleavage site, results in increased sIL-6R levels^11,12^ and is associated with a reduced risk of coronary heart disease^13,14^. The reason for this might be an anti-inflammatory effect on an increased buffer capacity of sIL-6R in combination with soluble gp130 (sgp130), which can bind and thus neutralize low levels of systemic IL-6^15,16^.

Soluble cytokine receptors can be generated by different molecular mechanisms^17^. For the sIL-6R, alternative splicing of the IL-6R pre-mRNA, which results in the excision of the exon encoding the transmembrane region^18^, and proteolytic cleavage of the membrane-bound IL-6R have been described^19^. Different proteases have been implicated in sIL-6R generation, e.g. neutrophil-derived serine proteases^20^ or meprins^21^. However, most work done to date has concentrated on the two metalloproteases ADAM10 and ADAM17. ADAM17 can be activated by different stimuli to provoke IL-6R cleavage, including the phorbol ester PMA^19,22^, cellular cholesterol depletion^23^ or apoptosis^24^. ADAM10 cleaves the IL-6R constitutively, but can also be stimulated to do so, e.g. by the ionophore ionomycin or via activation of the purinergic P2X7 receptor^25–27^. We have recently shown that the majority of about 85% of sIL-6R in the human circulation is generated via proteolysis, whereas the remaining ~ 15% are the result of alternative splicing^28^. The C-terminus of the proteolysis-derived sIL-6R ends with Pro-355, which means that the protease that cleaves the IL-6R in vivo uses the cleavage site between Pro-355 and Val-356. We have further demonstrated that ADAM17 uses exactly this cleavage site in vitro and that mutation of Val-356 is sufficient to generate an IL-6R variant that is not cleaved by ADAM17^28^. However, sIL-6R serum levels are unaltered in hypomorphic ADAM17^ex/ex^ mice, suggesting that another protease besides ADAM17 is responsible for the generation of sIL-6R serum levels under physiological conditions.

Cysteine cathepsins are a group of proteases with 11 members in humans^29^. Traditionally viewed as important degrading enzymes present in lysosomes, it is becoming more and more evident that cysteine cathepsins have crucial additional roles that are not restricted to the lysosome. Some family members are also present in other cellular compartments and even secreted as active proteases into the extracellular space^29,30^. One of the secreted family members is cathepsin S (CTSS), which is predominantly expressed in antigen-presenting cells^31^. Secreted CTSS is able to cleave membrane-bound substrates, e.g. the chemokine fractalkine^32^, which is due to the fact that CTSS, in contrast to most other cathepsins, is biologically activate at a neutral pH and thus does not require an acidic environment^33^.

In this study, we identify CTSS as a novel protease that can cleave the IL-6R. The sIL-6R generated by CTSS is biologically active and able to induce IL-6 trans-signaling. However, CTSS does not use the same cleavage site as ADAM17 and does not contribute to the generation of sIL-6R steady state levels in mice.

## Results

### Identification of candidate proteases for IL-6R cleavage based on the cleavage site

**Figure 1:**
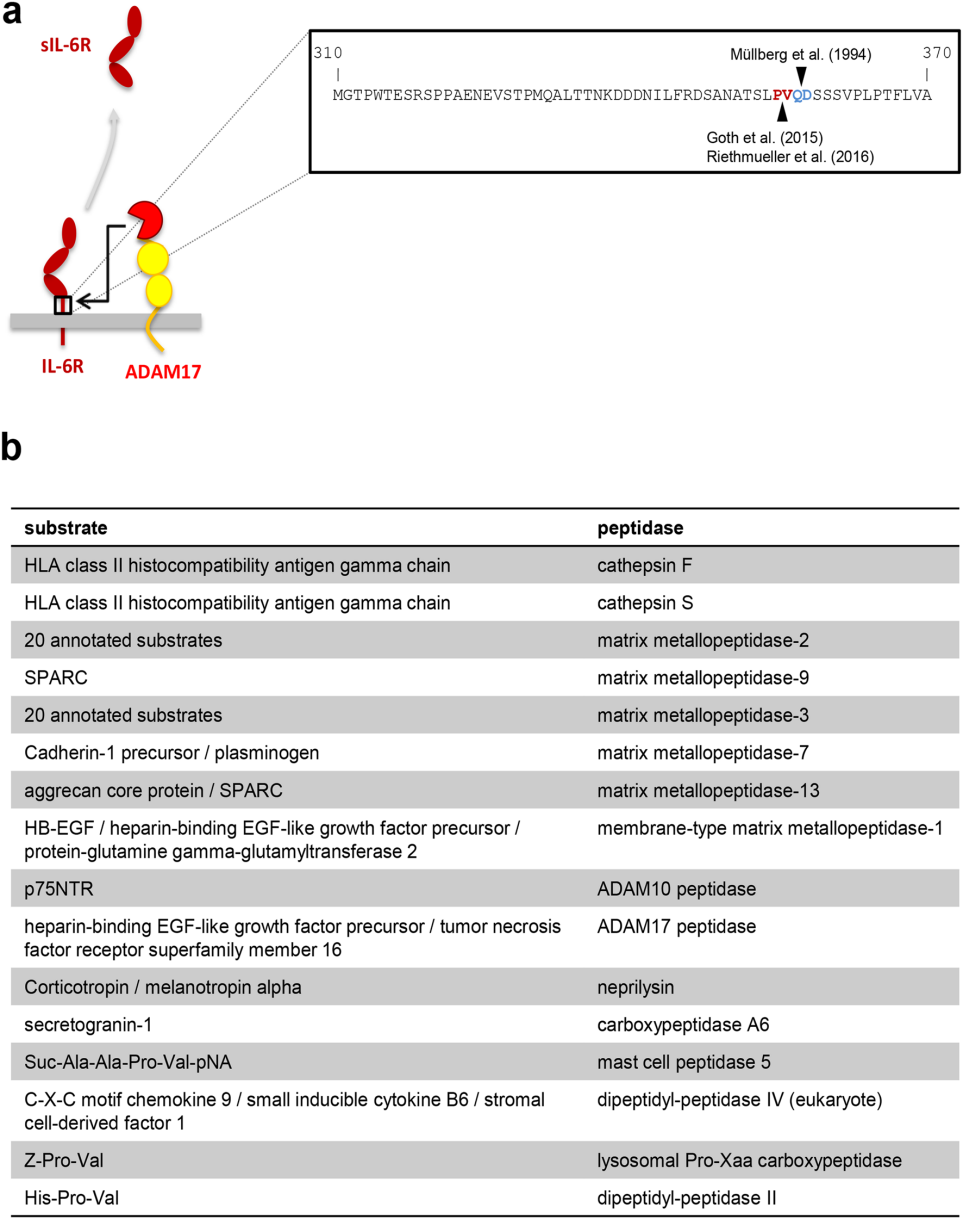
Identification of candidate proteases for IL-6R cleavage based on the cleavage site. **(a)** Schematic representation of sIL-6R generation via IL-6R proteolysis by ADAM17. The amino acids comprising the stalk region (from Met-310 to Ala-370) are depicted in one letter code in the inset on the right side. Previously described IL-6R cleavage sites attributed to ADAM17 are indicated. **(b)** Candidate proteases that could cleave the IL-6R between Pro-355 and Ala-356 based on the annotations found in the MEROPS database. For details see *Materials and Methods*.

The IL-6 trans-signaling pathway is initiated by cleavage of the membrane-bound IL-6R. Initial studies described a responsible protease that was activated by the phorbol ester PMA in a protein kinase C (PKC)-dependent manner^19^, which was later identified as ADAM17^34,35^. The used cleavage site was originally identified to be located between Gln-357 and Asp-358 within the so-called stalk region of the IL-6R close to the plasma membrane (Fig. 1a and^36^). Recent studies, however, could not reproduce this finding, but rather determined the ADAM17 cleavage site to be located between Pro-355 and Val-356 (Fig. 1a and^28,37^). We determined the correct cleavage site of the IL-6R by ADAM17 via mass spectrometry (MS) analyses of precipitated sIL-6R from human serum and identified a C-terminal peptide of the sIL-6R that originated from proteolytic cleavage and ended with the residue Pro-355. We obtained the same result when we stimulated HEK293 cells overexpressing IL-6R with PMA and analyzed precipitated sIL-6R via MS. Furthermore, mutation of the Pro-355/Val-356 cleavage site abrogated ADAM17-mediated IL-6R cleavage. Thus, we conclude that the exact position of the ADAM17 cleavage site in the IL-6R is at Pro-355^28^.

These results suggested that ADAM17 is the responsible protease that generates sIL-6R levels in vivo. However, the fact that the cleavage site, which ADAM17 uses in vitro is the same cleavage site that is used in vivo does not necessarily prove a causative role for ADAM17. Indeed, sIL-6R levels in hypomorphic ADAM17^ex/ex^ mice are unaltered compared to wild-type controls^25^. Thus, we hypothesized that a different protease cleaves the IL-6R in vivo that simply uses the same IL-6R cleavage site as ADAM17 does in vitro. In order to identify candidate proteases, we searched the MEROPS database^38^ for human proteases that are known to cleave their substrates between a proline and a valine residue. This search revealed 16 proteases (Fig. 1b), among them the known IL-6R sheddases ADAM17 and ADAM10^23^. The other candidates are two cathepsins, 5 matrix metalloproteases (MMPs), one matrix-type MMP (MT-MMP) and the proteases neprilysin, carboxypeptidase A6, mast cell peptidase 5, dipeptidyl-peptidase IV, lysosomal Pro-Xaa carboxypeptidase and dipeptidyl-peptidase II (Fig. 1b). Importantly, this approach does neither take into consideration the cellular localization nor the cell-type specific expression of the proteases but is solely based on the cleavage site preferences. For example, dipeptidyl-peptidases II and IV, carboxypeptidase A6 and lysosomal Pro-Xaa carboxypeptidase are exopeptidases and therefore unable to release the ectodomain of transmembrane proteins, which excludes them as the sought-after IL-6R protease. Thus, it is expected that not all of the proteases are indeed IL-6R sheddases.

### Expression profile of cathepsin S

**Figure 2:**
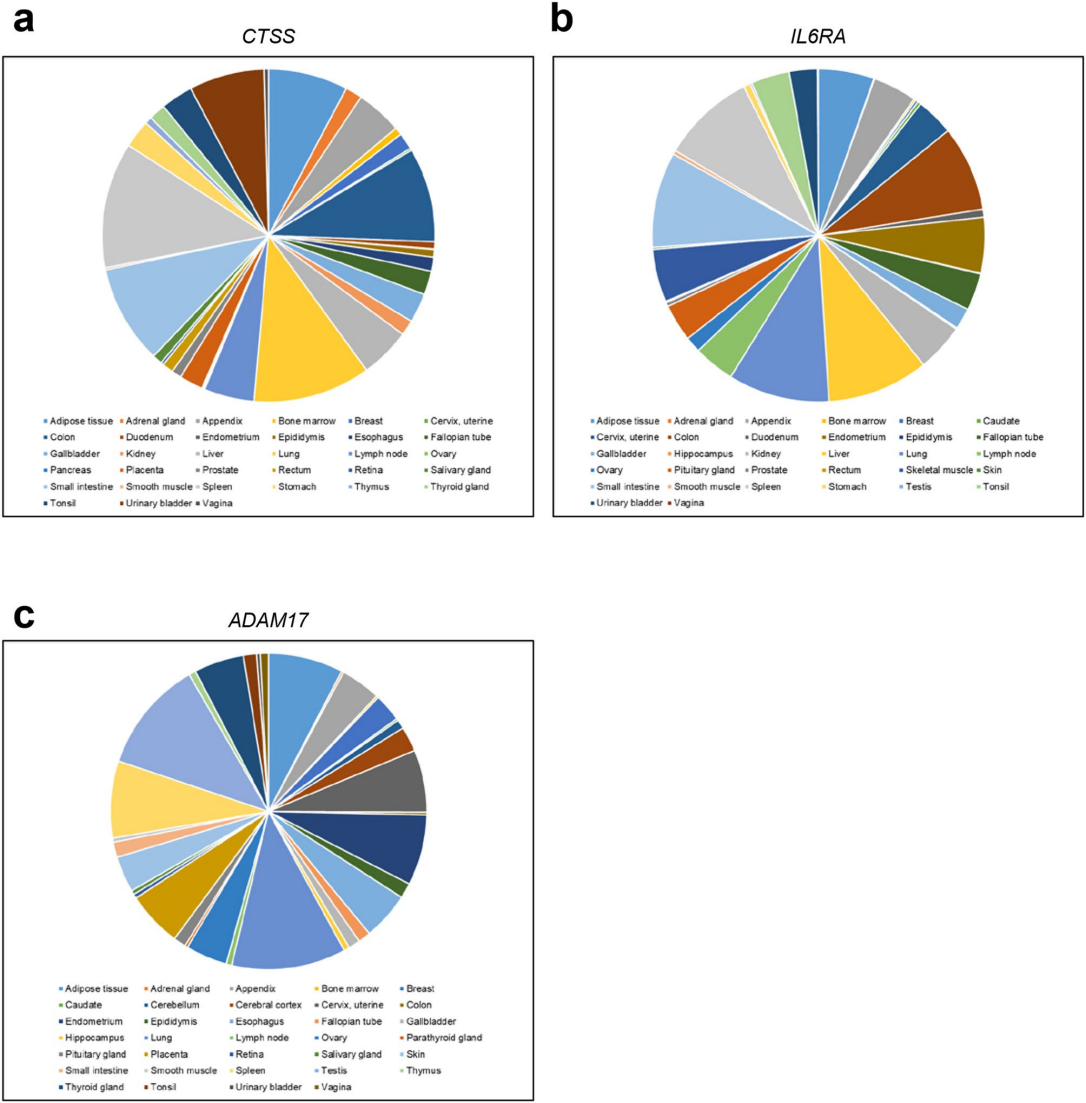
Expression profile of human *CTSS*, *IL6RA* and *ADAM17*. The pie charts show the twenty tissues with the highest expression of **(a)** CTSS, **(b)** IL6RA and **(c)** ADAM17. Expression data were analyzed as described previously^44^.

In order to validate whether our search approach based on the cleavage site (Fig. 1b) resulted at all in proteases that can cleave the IL-6R, we chose cathepsin S (CTSS) for further investigation. The reasons for investigating CTSS were that (1) the annotated substrate in the MEROPs database was a protein and not just a peptide (Fig. 1b) and (2) CTSS has been linked previously not only to inflammatory processes^39,40^, but also directly to IL-6^41,42^. For an IL-6R protease, one would expect that it is expressed in similar cell types as the IL-6R. In order to determine this, we analyzed mRNA expression of CTSS, IL-6R and ADAM17 in different human tissues. For this, we used quantitative expression data from the human protein atlas (v18.proteinatlas.org)^43^ and a scoring system developed by Düsterhöft et al.^44^ (Fig. 2a–c). Intriguingly, expression of CTSS and IL-6R highly correlated (Pearson Correlation 0.439, *p* = 0.003), and the same was true for ADAM17 and IL-6R (Pearson Correlation 0.716, *p* < 0.001) (Table 1). Thus, the expression pattern of CTSS and IL-6R open up the possibility that CTSS might indeed be a protease that could cleave the IL-6R.

**Table 1:** Matrix of correlations.

	CTSS	IL6RA	ADAM17
**CTSS**			
Pearson correlation	1.000		
Sig. (2-tailed)	–		
N	60		
**IL6RA**			
Pearson correlation	0.439**	1.00	
Sig. (2-tailed)	0.003	–	
N	45	60	
**ADAM17**			
Pearson correlation	0.512**	0.716**	1.00
Sig. (2-tailed)	0.001	0.000	–
N	39	39	60

**Correlation is significant at the 0.01 level (2-tailed).

### IL-6R peptide cleavage by cathepsin S and ADAM17

**Figure 3:**
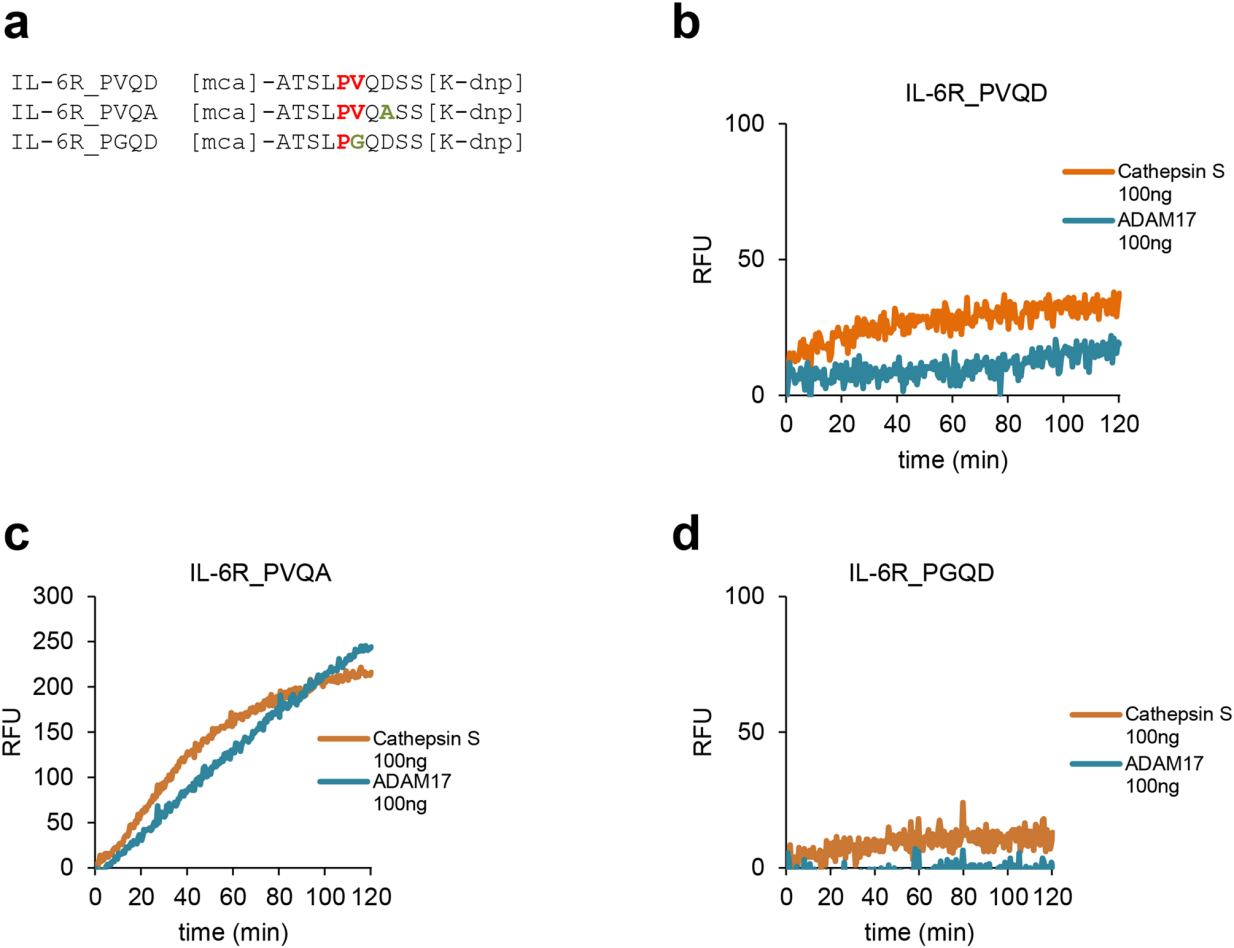
IL-6R peptide cleavage by Cathepsin S and ADAM17. **(a)** Sequences of the three peptides that were used in the experiments. IL-6R_PVQD corresponds to the wild-type sequence of the IL-6R, IL-6R-PVQA corresponds to an IL-6R containing the SNP rs2228145^10^ and IL-6R_PGQD corresponds to an IL-6R variants previously shown not to be cleaved by ADAM17^28^. **(b–d)** Proteolysis of the three peptides by recombinant Cathepsin S (100 ng) and recombinant ADAM17 (100 ng) as measured via an increase in fluorescence over 120 min.

Despite an initial report that the recombinant catalytic domain of ADAM17 is not capable of cleaving a peptide corresponding to a part of the IL-6R stalk region containing its cleavage site^45^, we^28^ and others^37^ have reported recently that such peptides are indeed processed at the correct Pro/Val cleavage site. Importantly, however, the IL-6R peptide is still a rather bad substrate for recombinant ADAM17, especially compared to corresponding peptides of TNFα, even after long incubation times^28^. We reasoned that recombinant CTSS should behave similarly and thus employed a peptide cleavage assay using three different internally quenched peptides containing the MCA/DNP pair, which emit fluorescence once the peptide is cleaved by a protease (Fig. 3a). The first peptide, named IL-6R_PVQD, consists of ten amino-acid residues of the wild-type IL-6R with the Pro/Val cleavage site in its center. This peptide is cleaved slowly by recombinant ADAM17 (Fig. 3b), which is consistent with our previous data^28^. Importantly, recombinant CTSS was also able to cleave the IL-6R_PVQD peptide (Fig. 3b). Next, we analyzed cleavage of the IL-6R_PVQA peptide. Here, the aspartic acid residue at position 8 (corresponding to position P3′ of the cleavage site) was replaced with an alanine residue (Fig. 3a). This peptide corresponds to an IL-6R variant encoded by the single nucleotide polymorphism (SNP) rs2228145, which leads to the aforementioned exchange of Asp358Ala of the IL-6R and results in increased sIL-6R levels^11,12^ due to enhanced proteolytic cleavage^10,28^. In line with this and compared to the IL-6R_PVQD peptide (Fig. 3b), the IL-6R_PVQA peptide was cleaved more rapidly by ADAM17 and CTSS as illustrated by an increase in fluorescence over time (Fig. 3c). As we have previously shown that mutation of the valine residue within the IL-6R cleavage site abrogated ADAM17-mediated cleavage^28^, we used lastly the peptide IL-6R_PGQD, which showed indeed strongly reduced cleavage by both ADAM17 and CTSS (Fig. 3d). In summary, our peptide cleavage data not only show that CTSS can cleave a peptide containing the IL-6R cleavage site, but also that CTSS behaves remarkably similar to ADAM17 regarding this cleavage site.

### Cathepsin S generates a biologically active sIL-6R independent of ADAM10 and ADAM17

**Figure 4:**
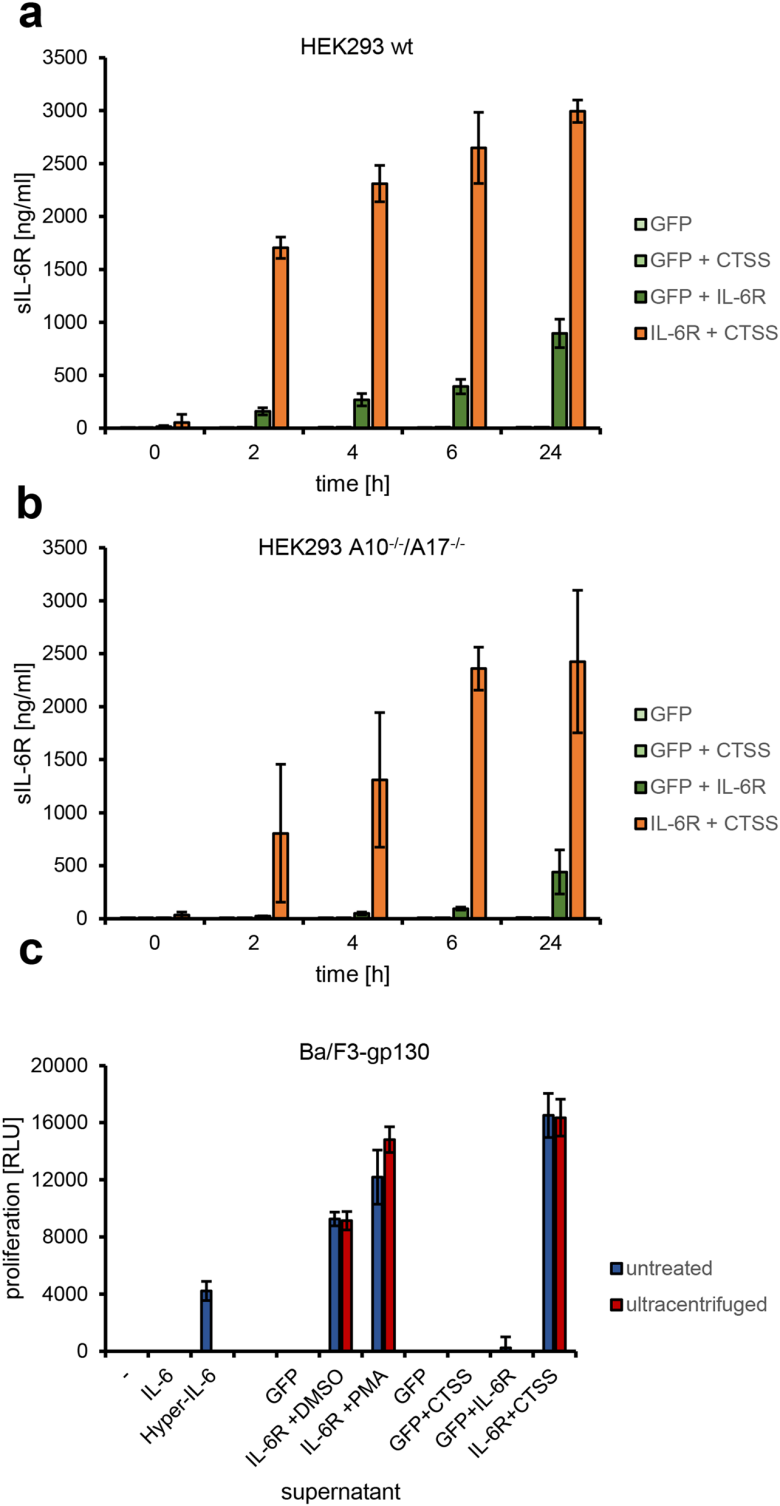
Cathepsin S generates a biologically active sIL-6R independent of ADAM10 and ADAM17. **(a)** HEK293 cells were transiently transfected with expression plasmids encoding GFP and CTSS, GFP and IL-6R, IL-6R and CTSS or only with an expression plasmid encoding GFP. The medium was exchanged 24 h after transfection and the conditioned medium harvested after different periods of time (0–24 h). The amount of sIL-6R in the medium was determined by ELISA (mean ± SD). **(b)** The experiment was performed as described for panel (**a**), but HEK293-ADAM10^−/−^/ADAM17^−/−^ cells were used instead. **(c)** In order to investigate whether the sIL-6R generated by CTSS was biologically active, equal amounts of Ba/F3-gp130 cells were stimulated with recombinant proteins and/or supernatants as indicated. Cell proliferation as measured by cell viability was determined 48 h later. Red colored bars indicate supernatant after ultracentrifugation, whereas the blue bars indicate supernatant without ultracentrifugation.

In order to investigate whether CTSS would also be able to release sIL-6R from cells, we transfected HEK293 cells and therefore overexpressed IL-6R together with either CTSS or GFP. We exchanged the medium 24 h after transfection and harvested the conditioned medium after different periods of time (0–24 h). As shown in Fig. 4a, HEK293 cells transfected with IL-6R and GFP released sIL-6R over time as measured by ELISA, consistent with previous findings that ADAM10 constitutively cleaves IL-6R^23,25,26^. This was three-fold increased when IL-6R and CTSS were both expressed in HEK293 cells, suggesting that CTSS was indeed able to perform IL-6R cleavage (Fig. 4a). Cells expressing either GFP alone or GFP in combination with CTSS served as control and did not release sIL-6R (Fig. 4a). To prove that the released sIL-6R resulted from direct cleavage by the expressed CTSS and was not indirectly released by one of the known IL-6R sheddases, we repeated the experiment using HEK293 cells deficient for ADAM10 and ADAM17 (HEK293-ADAM10^−/−^/ADAM17^−/−^), which we have described previously^27^. We detected strongly reduced sIL-6R release when these cells were transfected with IL-6R and GFP compared to HEK293 wt, but unaltered sIL-6R release when IL-6R and CTSS were co-expressed (Fig. 4b). These results show that ADAM10 and ADAM17 are not involved in sIL-6R generation by CTSS and indicate a direct proteolysis of the IL-6R by CTSS.

In order to investigate whether the sIL-6R generated by overexpressed CTSS was biologically active, we made use of the pre-B cell line Ba/F3-gp130. These cells proliferate in the presence of IL-6/sIL-6R complexes and undergo apoptosis otherwise^46^. Ba/F3-gp130 proliferated with supernatant containing sIL-6R cleaved by endogenous ADAM10 (IL-6R + DMSO), endogenous ADAM17 (IL-6R + PMA) and overexpressed CTSS (IL-6R + CTSS) when combined with recombinant IL-6 (Fig. 4c). This indicates that the sIL-6R generated by CTSS is indeed biologically active. However, IL-6R can also be released from cells on microvesicles^47^, and it might be possible that CTSS expression does not directly lead to IL-6R cleavage, but rather to the stimulation of the release of microvesicles containing IL-6R. In order to exclude this possibility, we performed ultracentrifugation of the supernatants, which would remove microvesicles but keep sIL-6R derived from proteolysis. As shown in Fig. 4c, ultracentrifugation did not reduce proliferation of Ba/F3-gp130 cells, which is consistent with our findings that CTSS directly cleaves the IL-6R.

### Cathepsin S uses a different IL-6R cleavage site than ADAM17

**Figure 5:**
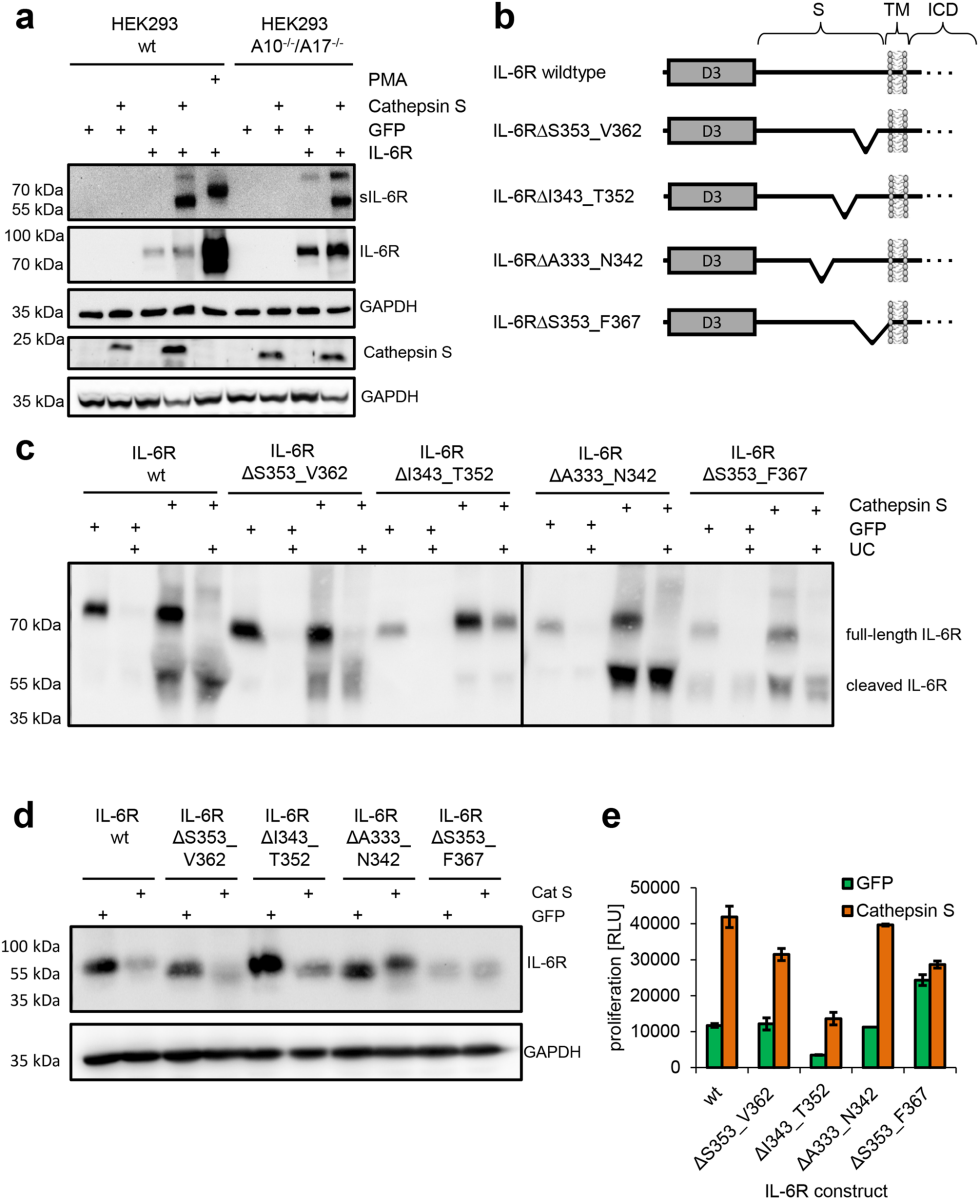
Cathepsin S uses a different IL-6R cleavage site than ADAM17. **(a)** The experiment was performed as described in the legends to panels Fig. 4a,b, but the sIL-6R was precipitated from the supernatant and visualized by western blotting. Furthermore, HEK293 cells transiently transfected with an expression plasmid encoding IL-6R were stimulated with PMA for 120 min, which activates ADAM17 and induces IL-6R proteolysis. The cells were lysed and also visualized by western blotting. GAPDH was determined to verify equal protein loading. Overexpressed Cathepsin S was visualized by Western blotting in an experiment that was conducted similarly, and GAPDH was determined to verify equal protein loading. **(b)** Schematic representation of the different IL-6R constructs that were used to map the cleavage site used by CTSS. The IL-6R constructs have been described previously^26^. **(c)** HEK293 cells were transiently transfected with expression plasmids encoding IL-6R in combination with either GFP or CTSS. The experiment was performed as described in the legend of panel (**a**), but before precipitation the supernatants were split in half and either ultracentrifuged or left untreated. **(d)** Western blot of the cell lysates corresponding to the supernatants shown in panel (**c**). GAPDH was determined to verify equal protein loading. **(e)** The experiment was performed as described in the legend to Fig. 4c. Supernatants of HEK293 cells transiently transfected with expression plasmids encoding the different IL-6R variants depicted below the bar diagram in combination with either CTSS or GFP were used.

Having shown that CTSS is able to release a biologically active sIL-6R, we sought to investigate whether CTSS would indeed use the previously identified cleavage site between Pro-355 and Val-356. We again transiently transfected HEK293 with different combinations of expression plasmids encoding IL-6R, CTSS and GFP (Fig. 5a). When we precipitated proteins from the supernatant of cells overexpressing IL-6R and CTSS, we detected two bands of different molecular weights around 80 kDa and 55 kDa. We concluded that the smaller sIL-6R derived probably from proteolysis by CTSS, while the larger IL-6R could be the full length variant on microvesicles that we had described previously^47^. Intriguingly, when we analyzed the supernatant of IL-6R-expressing HEK293 cells that had been stimulated with PMA to induce sIL-6R generation by ADAM17, the ADAM17-generated sIL-6R had a considerable higher molecular weight compared to the sIL-6R generated by CTSS (Fig. 5a). This suggests that overexpressed CTSS and endogenous ADAM17 do not use the same cleavage site when cleaving the full-length IL-6R protein. Importantly, we observed the same pattern of sIL-6R variants in the supernatant of HEK293-ADAM10^−/−^/ADAM17^−/−^ cells, which further underlines that sIL-6R generation is mediated directly by CTSS and does not involve ADAM10 and ADAM17 (Fig. 5a).

We have previously described different IL-6R variants that carry deletions within the stalk region^26^. In order to determine the region in which CTSS cleaves the IL-6R, we chose IL-6R∆S353_V362, IL-6R∆I343_T352 and IL-6R∆A333_N342, which lack consecutive stretches of 10 amino acid residues, and IL-6R∆S353_F367, which lacks the 15 amino acid residues adjacent to the plasma membrane (Fig. 5b). We transiently expressed IL-6R wild-type and the four IL-6R variants either together with GFP or with CTSS in HEK293 cells and analyzed sIL-6R generation by Western blot (Fig. 5c). For all IL-6R variants, we detected an IL-6R species in the supernatant with roughly the same molecular weight as the full-length construct found in the cell lysate (Fig. 5d). This sIL-6R was seen independent of CTSS co-expression and disappeared completely after ultracentrifugation of the supernatant, which is consistent with an IL-6R released on microvesicles. The smaller sIL-6R species only present when CTSS was co-expressed did not disappear after ultracentrifugation, indicative of soluble proteins derived from proteolysis (Fig. 5c). The cleavage product produced by overexpressed CTSS was clearly visible for all tested IL-6R variants with the exception of IL-6R∆I343_T352, for which only a faint band could be detected (Fig. 5c). In line with this, conditioned supernatant derived from transfected HEK293 cells resulted in the weakest proliferation of Ba/F3-gp130 cells when supernatant from IL-6R∆I343_T352 transfected cells was combined with recombinant IL-6 (Fig. 5e). We concluded from these experiments that CTSS and ADAM17 do not use the same cleavage site and that overexpressed CTSS instead uses a cleavage site further N-terminal between Ile-343 and Thr-352.

### Cathepsin S does not contribute to constitutive sIL-6R generation in vivo

**Figure 6:**
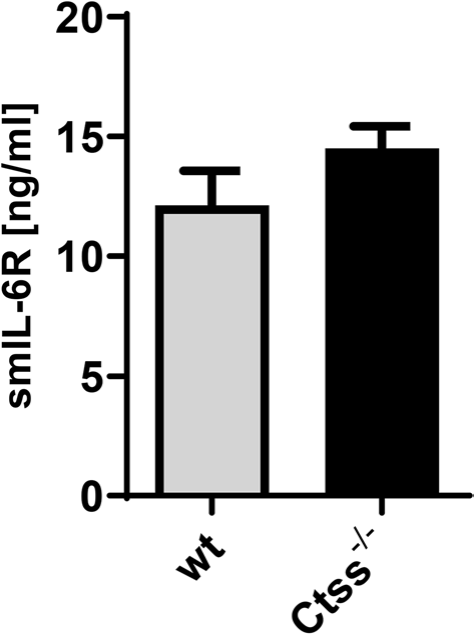
Cathepsin S does not contribute to constitutive sIL-6R generation in vivo. Levels of sIL-6R were determined via ELISA in serum samples from three wild-type and three *Ctss*^−/−^ mice (mean ± SD).

Serum levels of sIL-6R in humans are in the range of 20–80 ng/ml and in mice between 5 and 20 ng/ml. We have previously shown that in humans, the majority of sIL-6R is generated by proteolysis^28^ and that serum levels in ADAM17^ex/ex^ mice are unchanged compared to wild-type animals^25^. To determine whether CTSS contributes to sIL-6R levels in mice, we analyzed serum samples of *Ctss*^*−/−*^ mice. Compared to wild-type controls, we found no difference in sIL-6R serum levels (Fig. 6), which excludes a major role of CTSS in the generation of the steady state sIL-6R levels in mice.

## Discussion

The cytokine IL-6 is an important therapeutic target in inflammatory diseases, but possesses a very complex biology, which makes the development of therapeutic compounds without unwanted adverse effects challenging^3^. This is mostly due to the fact that IL-6 can signal via membrane-bound and soluble variants of the IL-6R and that the so-called trans-signaling of IL-6 is responsible for the pro-inflammatory functions of the cytokine. Therefore, a selective inhibition of signaling via the sIL-6R, which would leave classic signaling via the membrane-bound IL-6R untouched and preserve e.g. the hepatic acute phase response, holds the promise to be a next-generation therapeutic with a superior safety profile compared to the total blockade of IL-6 activity achieved with anti-IL-6 or anti-IL-6R antibodies that are already in clinical use^3^.

Several inflammatory stimuli are known that lead to the cleavage of the IL-6R at the plasma membrane and an increase in local sIL-6R levels, thus fostering IL-6 trans-signaling. These include apoptosis^24^, listeria monocytogenes infection^48^ or the activation of Toll-like receptor 2^49^ or the T cell receptor^50^. In all these contexts, the protease that cleaves the IL-6R upon stimulation has been identified as ADAM17, using specific chemical inhibitors and genetically modified mice, which makes it easy to assume that ADAM17-mediated IL-6R proteolysis represents a major mechanism for sIL-6R generation under inflammatory conditions.

However, how the steady state sIL-6R serum levels, which are present in mice and humans under normal physiological conditions, are generated is less well understood. We have shown previously that alternative mRNA splicing accounts for ~ 15% and that proteolytic cleavage accounts for ~ 85% of the sIL-6R found in human serum^28^. We were able to map the used cleavage site precisely via MS analyses, which is located in close proximity to the plasma membrane within the so-called stalk region of the IL-6R between Pro-355 and Val-356. Intriguingly, ADAM17 uses exactly the same cleavage site in vitro, making it a likely candidate for producing also the steady state sIL-6R serum levels^28^. However, hypomorphic ADAM17^ex/ex^ mice, in which the serum levels of other ADAM17 substrates are significantly reduced^51^, show unaltered sIL-6R levels, which implies that either ADAM17 is not the responsible protease, or that in the absence of ADAM17, another protease takes over the cleavage of the IL-6R.

We have therefore attempted to identify further candidate proteases that might be involved in sIL-6R generation. Based on the known cleavage site, we have mined the MEROPS database and found several proteases that could potentially cleave the IL-6R. We have concentrated on cathepsin S (CTSS), because its cellular expression profile matches the one of the IL-6R, it can be secreted from cells and would therefore be able to access the IL-6R, and finally because it is known to be involved in inflammatory reactions^39,40^. CTSS behaved in our initial peptide cleavage assays like ADAM17 and was able to release a biologically active sIL-6R in cellular assays that could bind IL-6 and perform trans-signaling on cells that lack the membrane-bound IL-6R and would thus without the sIL-6R not react to IL-6 alone. However, two important findings made it unlikely that CTSS was the protease we searched for. Using different IL-6R stalk mutants, we could show that CTSS does not use the cleavage site between Pro-355 and Val-356 that we had determined previously, but rather cleaves the IL-6R at a position further N-terminal within the stalk region, which excludes that CTSS is involved in sIL-6R generation in humans. Secondly, the serum levels in healthy *Ctss*^*−/−*^ mice were unaltered compared to wild-type control mice, judging against an involvement of this protease in sIL-6R generation also in mice.

In conclusion, although we were able to identify a novel protease that can cleave the IL-6R and thus activate IL-6 trans-signaling, which might be important for example under inflammatory conditions in which CTSS is overexpressed, our data rule out a contribution of CTSS to the formation of the steady-state sIL-6R serum levels. Further research is needed to identify the responsible protease(s) for this.

## Methods

### Cells and reagents

HEK293 and HEK293-ADAM10^−/−^/ADAM17^−/−^ cells, which have been described previously^27^, were cultured in DMEM high-glucose culture medium (Gibco/Thermo Fisher Scientific, Waltham, MA, USA) supplemented with 10% fetal bovine serum, penicillin (60 mg/l), and streptomycin (100 mg/l). All cells were kept at 37 °C and 5% CO_2_ in a standard incubator with a water-saturated atmosphere. Cells were transiently transfected with the cationic polymer solution TurboFect Transfection Reagent (Thermo Fisher Scientific, Waltham, MA, USA). Expression plasmids encoding IL-6R and Cathepsin S have been described previously^25,52^. The protein kinase C activator phorbol-12-myristate-13-acetate (PMA) was purchased from Sigma-Aldrich (St. Louis, MO, USA). The peptides [mca]-ATSLPVQDSS[K-dnp] (IL-6R_PVQD), [mca]-ATSLPVQASS[K-dnp] (IL-6R_PVQA) and [mca]-ATSLPGQDSS[K-dnp] (IL-6R_PGQD) were synthesized by Genosphere Biotechnologies (Paris, France). The cytokine IL-6 was expressed and purified as described previously^53^, as well as the designer cytokine Hyper-IL-6^46,54^. The antibody 4–11 (IL-6R antibody) was also expressed and purified as described previously^24^. The GAPDH antibody was purchased from Cell Signaling Technology (Frankfurt/M., Germany), the polyclonal rabbit anti‑human Cathepsin S antibody (LS‑C348945) was from LSBio (Seattle, WA, USA), and the secondary antibodies mouse-HRP and rabbit-HRP were obtained from Dianova (Hamburg, Germany). The recombinant protease cathepsin S was purchased from R&D systems (Minneapolis, USA).

### Ectodomain shedding assay

HEK293 wt and HEK293-ADAM10^−/−^/ADAM17^−/−^ cells were transiently transfected with plasmids coding for GFP, CTSS and different IL-6R variants. Two days after the transfection, the medium was exchanged with 5 ml fresh serum-free DMEM and the cells were incubated for 24 h at 37 °C before supernatants and cells were collected. Cells were lysed and supernatants were either used directly for protein precipitation by 20% TCA or were ultra-centrifuged (60 min at 4 °C and 100,000 g) before use. Finally, precipitated supernatants and lysates were analyzed by semi-dry western immunoblotting, which was performed as described previously^28^.

### Ba/F3-gp130 cell viability assay

To analyze whether generated sIL-6R is biologically active, a cell viability assay was performed using Ba/F3-gp130 cells and the CellTiter Blue Viability Assay (Promega, Karlsruhe, Germany) following the manufacturer’s instructions. Ba/F3-gp130 cells were washed with PBS, resuspended in DMEM supplemented with 10% FCS and 1% PenStrep solution and 5000 cells in 50 µl cell culture medium were transferred in wells of a 96-well plate. 50 µl of conditioned medium from transfected HEK293 cells or, as control, fresh medium was added to the cells. The cells were incubated with IL-6 (25 ng/ml), Hyper-IL-6 (10 ng/ml) or without cytokine for 48 h at 37° and 5% CO_2_ before 20 µl Cell Titer Blue Viability Assay reagent was added. Fluorescence intensity (RLU; relative light units) was measured at λem = 590 nm using the Synergy HTX multimode reader (BioTek, Winooski, VT, USA). Fluorescence at time point 60 min was normalized by subtracting the fluorescence measured at time point 0 min.

### Quantification of human and murine sIL-6R

For the detection of murine sIL-6R in murine sera the Mouse IL-6R alpha DuoSet ELISA (R&D systems, Minneapolis, USA) was performed according to the user instructions by addition of 1:10 diluted sera. Levels of human sIL-6R in cell culture supernatant was analyzed using the DuoSet Human IL-6Rα ELISA kit (R&D systems, Minneapolis, USA) following the user instructions with slight adaptations. ELISA plates were coated with 50 µl capture antibody overnight, washed, and 50 µl of undiluted cell culture supernatant was added. Following, the samples were incubated with 50 µl of detection antibody before the enzymatic reaction was started using streptavidin–horseradish peroxidase and the peroxidase substrate BM blue POD (Roche, Mannheim, Germany). The reaction was stopped using 1.8 M sulfuric acid and the absorbance was read at 450 nm on a Tecan Spectra Rainbow plate reader (Tecan, Crailsheim, Germany).

All experiments were performed in accordance with relevant guidelines and regulations and approved by the responsible committee in Erlangen (Amt für Veterinärwesen und Gesundheitlichen Verbraucherschutz, approval #TS-6/12).

### Peptide cleavage assay

For the peptide cleavage assay, 10 µM of the three different quenched fluorogenic IL-6R peptides [mca]-ATSLPVQDSS[K-dnp], [mca]-ATSLPVQASS[K-dnp] or [mca]-ATSLPGQDSS[K-dnp] (synthesized by Genosphere Biotechnologies, Paris, France) were incubated with 100 ng of the proteases ADAM17 or Cathepsin S in a total volume of 100 µl in PBS. Fluorescence was measured at λem = 405 nm and λex = 320 nm at 37 °C with a spectrophotometer (Tecan) every 30 s for 120 min. The increase in relative fluorescence units (RFUs) after 120 min was normalized to fluorescence at 0 s.

### In silico analyses

Candidate proteases were identified with the use of the MEROPS database (https://www.ebi.ac.uk/merops/)^38^. Here, the function “Search by specificity” was used to search for proteases that are known to cleave substrates with a proline residue at the P1 and a valine residue at the P1′ position. All non-human proteases were removed from the search results. The expression profiles shown in Fig. 2 were generated using an approach described previously^44^.

### Statistical analyses

Statistical analyses were performed with the Statistical Package for the Social Sciences (IBM SPSS Statistics for Windows, Version 25.0. Armonk, NY, USA). Pearson product-moment correlation coefficients were used to detect linear correlations between *CTSS, IL6RA*, and *ADAM17*. A high positive/negative correlation, ranging from − 1 to + 1, implies that high values of one measure correspond to high (positive correlation) or low (negative correlation) values of the other measure.

### Data presentation

Unless stated otherwise, data are presented as mean ± SD from at least three independent experiments. For western blots and Ba/F3-gp130 cell viability data, one experiment from at least three with similar outcome is shown.

## Data availability

No restrictions on the availability of materials or information apply. Requests for data and material should be addressed to the corresponding author (christoph.garbers@med.ovgu.de).

## Supplementary information

Supplementary Information.
