# Supplementary material for: Cathepsin S provokes interleukin-6 (IL-6) trans-signaling through cleavage of the IL-6 receptor in vitro
Source: Sci Rep. 2020 Dec 10;10:21612. doi: 10.1038/s41598-020-77884-4 (PMC7730449; doi:10.1038/s41598-020-77884-4)

Cathepsin S provokes interleukin-6 (IL-6) trans-signaling through cleavage of the IL-6 receptor in vitro

Charlotte M. Flynn[1], Yvonne Garbers[2], Stefan Düsterhöft[3], Rielana Wichert[1], Juliane Lokau[4], Christian H.K. Lehmann[5,6], Diana Dudziak[5,6], Bernd Schröder[7], Christoph Becker-Pauly[1], Stefan Rose-John[1], Samadhi Aparicio-Siegmund[1], and Christoph Garbers[4,*]

[1]Institute of Biochemistry, Kiel University, Kiel, Germany;
[2]Institute of Psychology, Kiel University, Kiel, Germany;
[3]Institute of Molecular Pharmacology, RWTH Aachen University, Aachen, Germany;
[4]Department of Pathology, Otto-von-Guericke-University Magdeburg, Medical Faculty, Magdeburg, Germany;
[5]Laboratory of Dendritic Cell Biology, Department of Dermatology, University Hospital Erlangen, Friedrich-Alexander University of Erlangen-Nürnberg, Erlangen, Germany;
[6]Deutsches Zentrum Immuntherapie (DZI) and Medical Immunology Campus Erlangen, Germany
[7]Institute for Physiological Chemistry, Medizinisch-Theoretisches Zentrum MTZ, Technische Universität Dresden, Fiedlerstraße 42, 01307, Dresden, Germany

[*]Correspondence to: Dr. Christoph Garbers (christoph.garbers@med.ovgu.de, +49 391 67-15488), Department of Pathology, Otto-von-Guericke-University Magdeburg, Medical Faculty, Magdeburg, Germany; Fax: +49 391 67-15818

Figure 5a

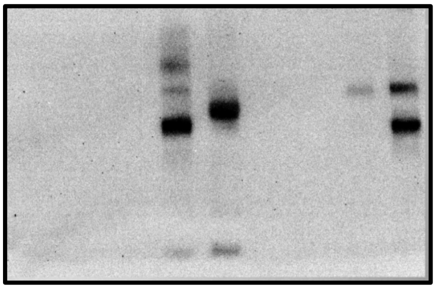

sIL-6R

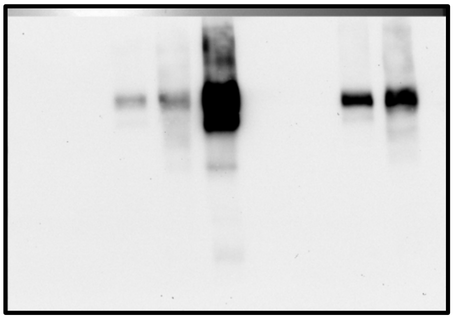

IL-6R

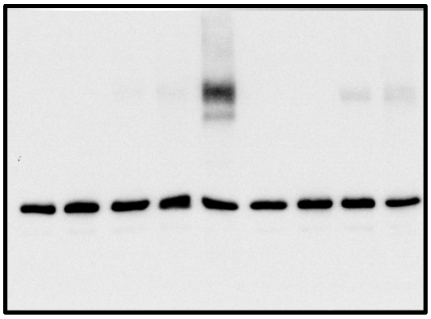

GAPDH

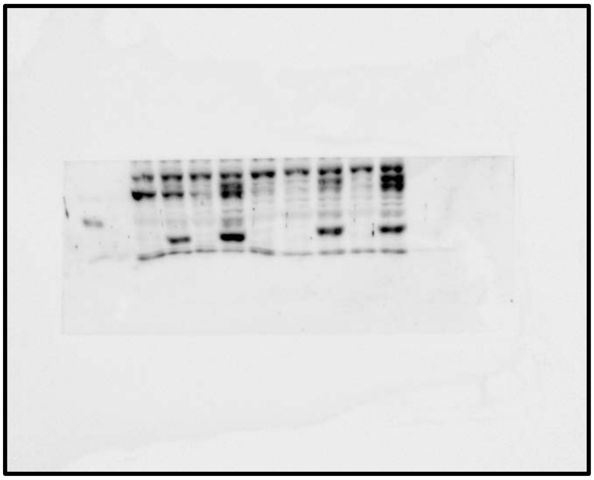

Cathepsin S

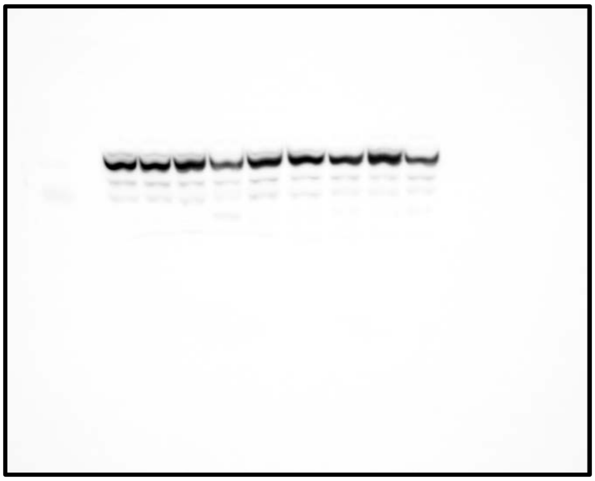

GAPDH

Figure 5c

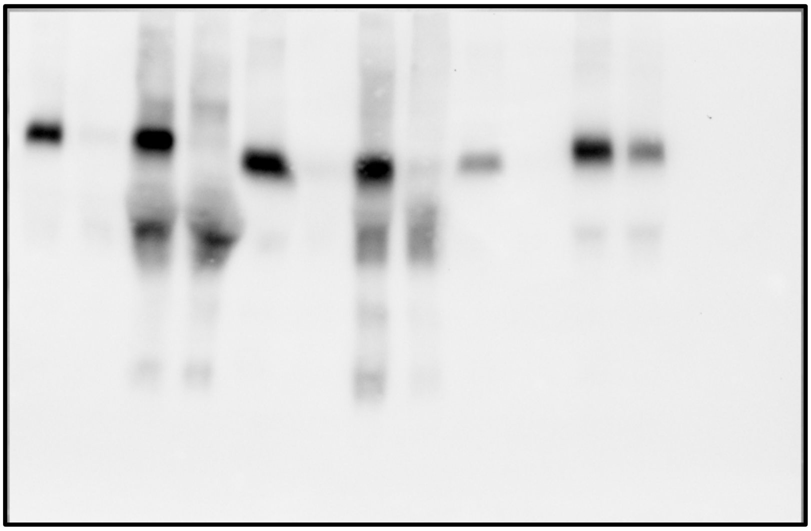

IL-6R

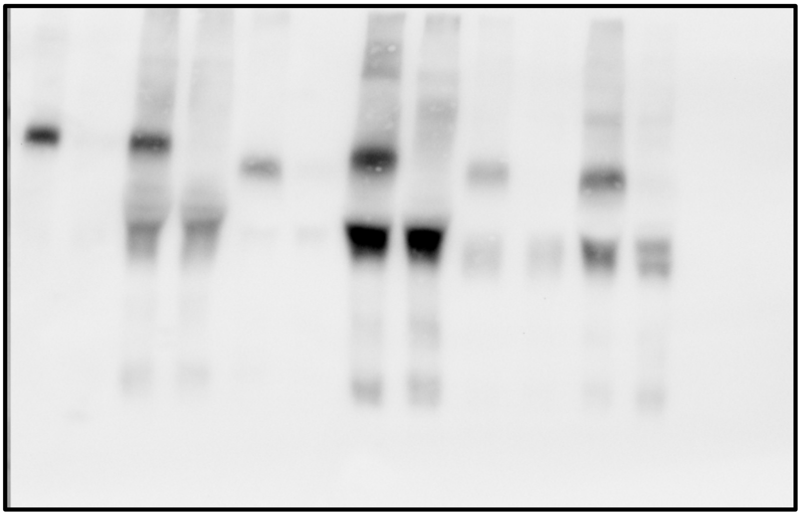

IL-6R

Figure 5d

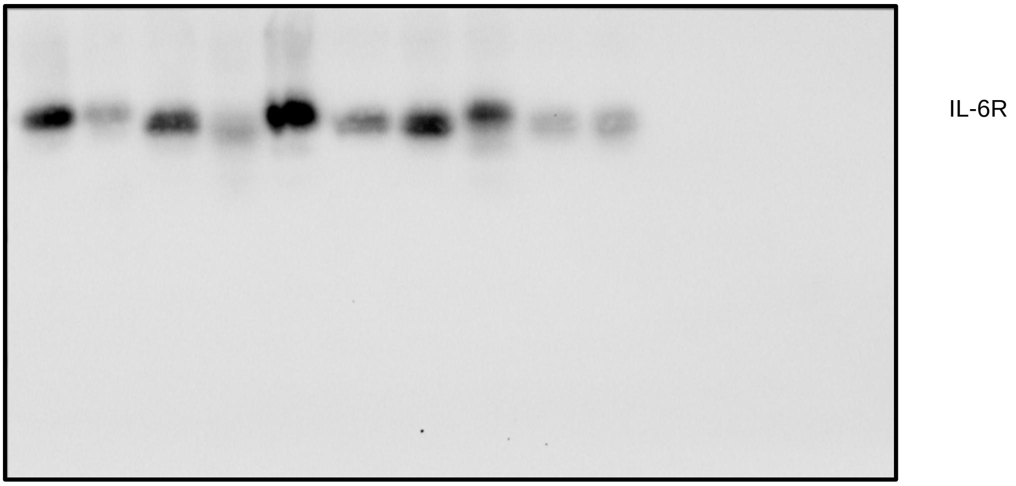

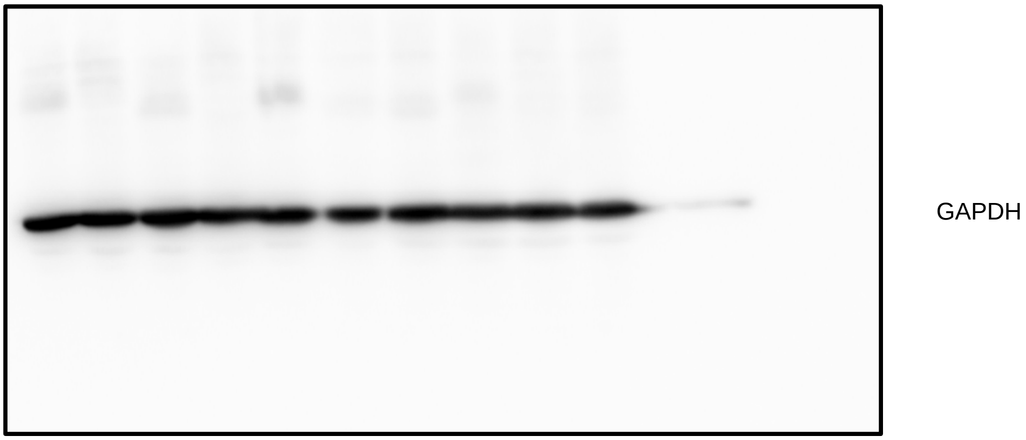

Supplement: Supplementary file 1 — Supplementary Information. [file 41598_2020_77884_MOESM1_ESM.pdf]
